# Transcriptional and Immunologic Correlates of Response to Pandemic Influenza Vaccine in Aviremic, HIV-Infected Children 

**DOI:** 10.3389/fimmu.2021.639358

**Published:** 2021-03-25

**Authors:** Lesley R. de Armas, Varghese George, Abdelali Filali-Mouhim, Courtney Steel, Anita Parmigiani, Coleen K. Cunningham, Adriana Weinberg, Lydie Trautmann, Rafick-Pierre Sekaly, Mark J. Cameron, Savita Pahwa

**Affiliations:** ^1^ Department of Microbiology and Immunology, University of Miami Miller School of Medicine, Miami, FL, United States; ^2^ Department of Neurosciences, CRCHUM University of Montreal, Montreal, QC, Canada; ^3^ Collaborative Genomics Center, Vaccine and Gene Therapy Institute, Port St. Lucie, FL, United States; ^4^ Department of Pediatrics, Duke University School of Medicine, Durham, NC, United States; ^5^ Departments of Medicine, Pathology, and Pediatric Infectious Diseases, University of Colorado School of Medicine, Aurora, CO, United States; ^6^ Vaccine and Gene Therapy Institute, Oregon Health and Science University, Portland, OR, United States; ^7^ Department of Pathology, Emory University School of Medicine, Atlanta, GA, United States; ^8^ Department of Population and Quantitative Health Sciences, Case Western Reserve University, Cleveland, OH, United States

**Keywords:** pandemic, influenza, vaccine, pediatric, HIV, microarray, systems vaccinology

## Abstract

People living with HIV (PWH) often exhibit poor responses to influenza vaccination despite effective combination anti-retroviral (ART) mediated viral suppression. There exists a paucity of data in identifying immune correlates of influenza vaccine response in context of HIV infection that would be useful in improving its efficacy in PWH, especially in younger individuals. Transcriptomic data were obtained by microarray from whole blood isolated from aviremic pediatric and adolescent HIV-infected individuals (4-25 yrs) given two doses of Novartis/H1N1 09 vaccine during the pandemic H1N1 influenza outbreak. Supervised clustering and gene set enrichment identified contrasts between individuals exhibiting high and low antibody responses to vaccination. High responders exhibited hemagglutination inhibition antibody titers >1:40 post-first dose and 4-fold increase over baseline. Baseline molecular profiles indicated increased gene expression in metabolic stress pathways in low responders compared to high responders. Inflammation-related and interferon-inducible gene expression pathways were higher in low responders 3 wks post-vaccination. The broad age range and developmental stage of participants in this study prompted additional analysis by age group (e.g. <13yrs and ≥13yrs). This analysis revealed differential enrichment of gene pathways before and after vaccination in the two age groups. Notably, *CXCR5*, a homing marker expressed on T follicular helper (Tfh) cells, was enriched in high responders (>13yrs) following vaccination which was accompanied by peripheral Tfh expansion. Our results comprise a valuable resource of immune correlates of vaccine response to pandemic influenza in HIV infected children that may be used to identify favorable targets for improved vaccine design in different age groups.

## Introduction

It is well established that very young, elderly and immune compromised individuals including people living with HIV (PWH) are at higher risk of influenza infection and related complications, underscoring the need for effective vaccination in these populations (1, 2). The Centers for Disease Control recommends seasonal influenza vaccination for all persons above six months of age (3), but seasonal vaccines have shown modest efficacy (4) and low antibody titers are generated in the elderly (over age 60 years) (5, 6) and in PWH (7). In particular, children and adolescents living with perinatally acquired HIV infection have impaired responses to vaccinations, including influenza vaccination, despite successful viral suppression by combination anti-retroviral therapy (ART) (8–10).

Influenza vaccination confers protection primarily *via* humoral immunity (11, 12). In response to natural infection, neutralizing antibodies are critical for blocking infection while cell-mediated immunity clears the virus (13, 14). Molecular and immunological factors contributing to protection induced by vaccines have been studied amply in recent years. Systems biology approaches have been used to evaluate immune responses to vaccines, e.g. yellow fever (15–17), meningococcus (18, 19), pneumococcal (18, 20) and influenza (21–23) and have been powerful tools for elucidating immunological correlates of vaccine responses. In the context of seasonal influenza vaccination, gene sets related to immunoglobulins, complement proteins, and cellular proliferation are strongly enriched in vaccine responders compared to non-responders 7 days post-vaccination (22). Ex vivo studies show that antibody-secreting B cells exhibit peak proliferation around day 7 post-vaccination (24–26), thereby validating transcriptomic analyses in vaccine biology. Based on gene signatures alone, transcriptomic analysis from pre-vaccination samples across multiple cohorts was used to predict response to influenza vaccination with accuracy above 83% (27). However, the majority of these studies focus on healthy, young adults leaving many questions still unanswered regarding PWH and other immune-compromised populations.

In 2009, the WHO declared the pandemic influenza A H1N1 swine-origin influenza virus a novel strain. Children were found to have no pre-existing immunity to the new strain but older adults (over age 60 years) had some degree of immunity attributed to cross reactivity to past influenza strains (28). A clinical trial (P1088) launched by the International Maternal Pediatric and Adolescent Clinical Trials (IMPAACT) Network evaluated safety and efficacy of a monovalent pandemic H1N1 (pH1N1) vaccine in perinatally HIV-1-infected children and adolescents (29). We utilized a systems biology approach to evaluate gene signatures from peripheral blood before and after pH1N1 vaccination in participants of the IMPAACT P1088 study with integration of serum antibody titer data from the same individuals. Multiple gene set enrichment databases were used to correlate gene expression patterns with antibody titers induced by vaccination and create this resource for this unique patient cohort. In light of the SARS-CoV-2 pandemic beginning in 2019, this study may have further relevance to the study of vaccine responses to novel antigens in children and adolescents living with HIV infection.

## Materials and Methods

### IMPAACT P1088 Clinical Study Participants and Immunogenicity Assessments

Specimens from the P1088 clinical trial “Safety of and Immune Response to an H1N1 Influenza Virus Vaccine in HIV Infected Children and Youth”, aged 4-24 years (n=40, mean age 13.7 yrs, 17 females and 23 males), were obtained from IMPAACT sites in the United States and Puerto Rico. All participants in the current study were HIV positive and receiving stable ART for at least 90 days before entry and had HIV RNA copies/ml ≤50. Other exclusion and inclusion criteria were described in the original study (29). In the trial, 155 participants received two doses (30ug) of 2009 Novartis influenza A (H1N1) monovalent vaccine separated by 21-28 days, each delivered as two 0.5 ml (15ug) injections into the thigh muscle. This study used blood samples collected pre-vaccination (baseline, BL) and 21-28 days post-first vaccination (visit 1, V1). Blood was processed for PBMC and plasma and an aliquot (2.5ml) was collected in PAXgene tubes and shipped overnight to the Miami IMPAACT laboratory at room temperature. Immunogenicity was determined by specific hemagglutination inhibition (HAI) titers in serum. The HAI assay was adapted from previously described methods (30).

### Microarray Experiments on Whole Blood

Total RNA was isolated using PreAnalytix PAXgene Blood RNA Isolation Kits (Qiagen), globin removed using GLOBINclear Kit (Ambion), and the quantity and quality of the RNA was confirmed using a NanoDrop 2000c (Thermo Fisher Scientific) and an Experion Electrophoresis System (BioRad). Samples (50 ng) were amplified using Illumina TotalPrep RNA amplification kits (Ambion). The microarray analysis was conducted using 750 ng of biotinylated complementary RNA hybridized to HumanHT-12_V4 BeadChips (Illumina) at 58°C for 20 h. The arrays were scanned using Illumina’s iSCAN. All microarray data is available under GEO reference number GSE167893.

### PBMC Culture and Flow Cytometry

Cryopreserved PBMC from BL and V1 were thawed and allowed to rest overnight at 37°C in culture medium (RPMI containing 10% FBS and pen/strep). For surface staining: PBMC were labeled with fluorescently-conjugated antibodies to human CD3, CD4, CD8, CD38, CD45RO, CXCR5 and HLA-DR. For 12 hr stimulation and intracellular cytokine staining: PBMC were cultured with or without 5ug/ml H1N1 (A/California/09) for 12hr at 37°C prior to the staining procedure. Data was acquired on BD Fortessa Instrument and analyzed using FlowJo software version 9.7.6 (TreeStar).

### Statistics

Quantile normalization, followed by a log2 transformation using the Bioconductor package LIMMA was applied to process microarrays. The LIMMA package was used to fit a linear model to each probe and perform (moderated) *t* tests or *F* tests on the groups being compared. To control the expected proportions of false positives, the FDR for each unadjusted *P* value was calculated using the Benjamini and Hochberg method implemented in LIMMA. Multidimensional scaling was used as a dimensionality reduction method in R to generate plots for evaluation of similarities or dissimilarities between datasets. For data mining and functional analyses, genes that satisfied a *p*-value (<0.05) with ≥ 1.3 fold change (up or down) were selected. The differentially expressed genes selected based on above criteria were mapped to ingenuity pathway knowledge base with different colors (red: up-regulated; blue: down-regulated). Significance of the association between the dataset and canonical pathway was measured in two ways (1): A ratio of the number of genes from the dataset that map to the pathway divided by the total number of genes that map to the canonical pathway; (2) over-representation analysis where Fisher’s test was used to calculate a *p*-value determining the probability that the association between the genes in the dataset and the canonical pathway is explained by chance alone.

## Results

### Baseline Molecular Profiles Are Associated With pH1N1 Responsiveness

**Figure 1:**
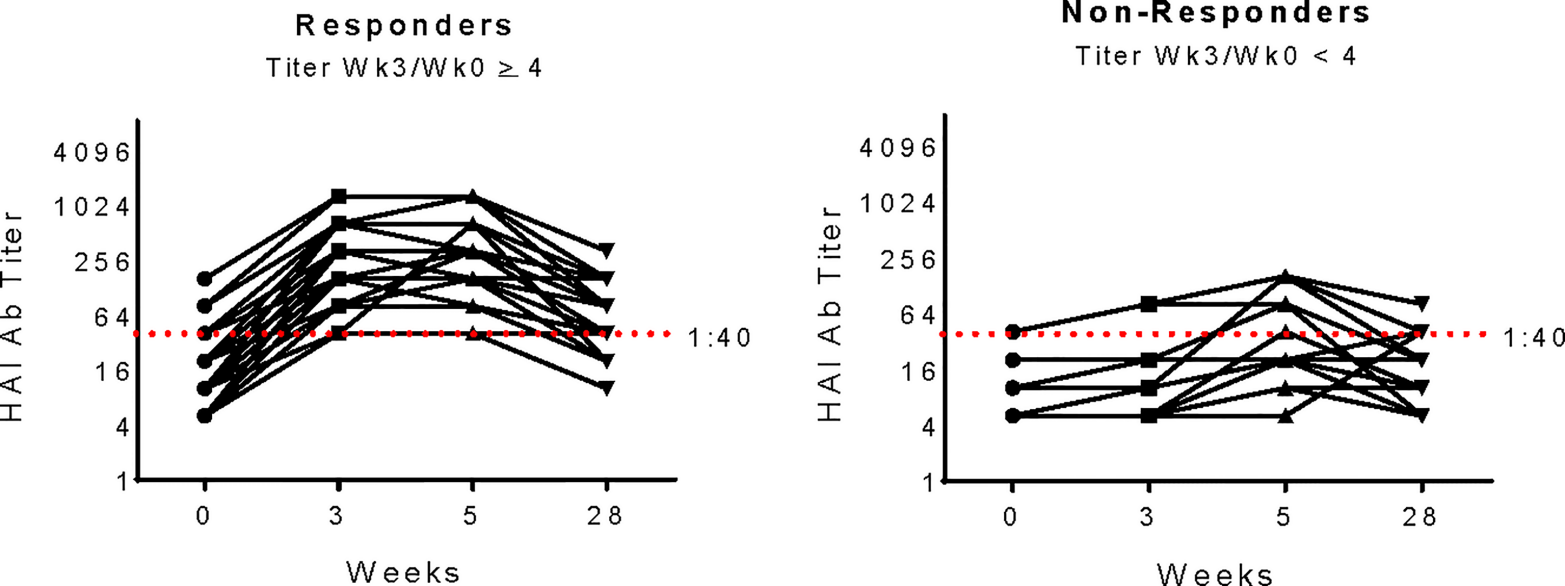
pH1N1 Serology for Study participants. pH1N1 Ab titers as determined by Hemagluttination Inhibition Assay (HAI) in study participants from Responder and Non-responder groups at each timepoint in the IMPAACT P1088 study. Responders (n=29, left panel) were defined as exhibiting a 4-fold increase at week 3 compared to week 0 and Non-responders (n=11right panel) failed to increase titers at least 4-fold between week 0 and 3. The red dashed line at titer 1:40 shows the accepted threshold for sero-protection.

The primary goal of our study was to identify gene expression signatures that correlated with immunogenicity of pH1N1 monovalent vaccine in PWH of younger age groups. Vaccine response was determined by measuring hemaglutination inhibition (HAI) titers in serum before and after immunization (**Figure 1**). High responders were distinguished by exhibiting a ≥ 4-fold increase at week 3 (visit 1, V1) compared to week 0 (baseline, BL), while low/non-responders failed to increase titer at least 4-fold between these timepoints (31).

**Figure 2:**
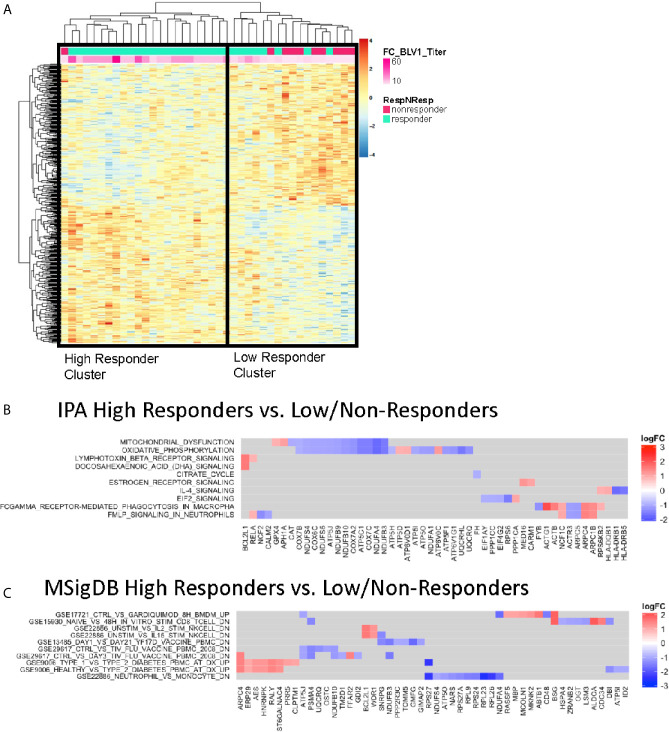
Baseline Enriched Molecular Pathways Associated with Antibody Titer Response. **(A)** Heatmap representation of the top 1,000 significantly correlated transcripts by regressing baseline gene expression from all participants against V1 pH1N1 titer. The expression intensities are represented using a blue-white-red color scale. Rows correspond to probes and columns correspond to profiled samples (p ≤ 0.01). High responder and Low/non responder clusters were compared and subjected to gene set enrichment analysis using the IPA database **(B)** and MSigDB immunologic signature model **(C)**.

To investigate gene expression profiles predictive of antibody response to vaccination, we performed regression analysis using microarray data from BL samples against fold change pH1N1 titers (V1/BL). Two-way hierarchical cluster analysis of the top genes correlating with responder status (**Supplementary Table S1**) divided participants into 2 distinct clusters (**Figure 2A**). Downstream gene set enrichment analysis (GSEA) analyses of differentially expressed genes was performed on participants in the clusters using Ingenuity Pathway Analysis (IPA) and the immunologic signature module from Molecular Signatures Database (MSigDB), the latter of which contains published, manually curated gene sets from the Gene Expression Omnibus (GEO) that represent cell types, states, and perturbations of the human and mouse immune system. We found that the Low/non-responder (LNR) group exhibited pathway enrichment in mitochondrial dysfunction, oxidative phosphorylation, cytokine signaling modulation (LTB, IL-4), macrophage signaling (Fc-gamma receptor-mediated phagocytosis), and EIF2 signaling (stress-related signaling) at BL compared to high responder (HR) group (**Figure 2B**). In MSigDB analysis several gene sets identified were derived from studies with Flu-vaccinated HIV-negative adults (GSE29617) (21) and day 21 yellow fever vaccine responses in human PBMCs (GSE13485) (**Figure 2C**). The gene *BCL21L* encoding the cell death inhibitor protein Bcl-2 like protein was highly enriched in HR. HR displayed gene signatures resembling pre-vaccination signatures from HIV-negative individuals (GSE29617_CTRL_VS_TIV_FLU_VACCINE_PBMC_ 2008). Some genes from this pathway (*ATP5J, UQCRQ, PSMA4, and NDUFB10*) overlapped with IPA analysis as members of the mitochondrial dysfunction and oxidative phosphorylation pathways. Overall, the BL gene expression data suggests that enrichment of mitochondrial or oxidative stress transcriptional pathways at the time of vaccine administration may confer poor responses to vaccination.

### Post-Vaccination Molecular Profiles Associated With pH1N1 Responsiveness

**Figure 3:**
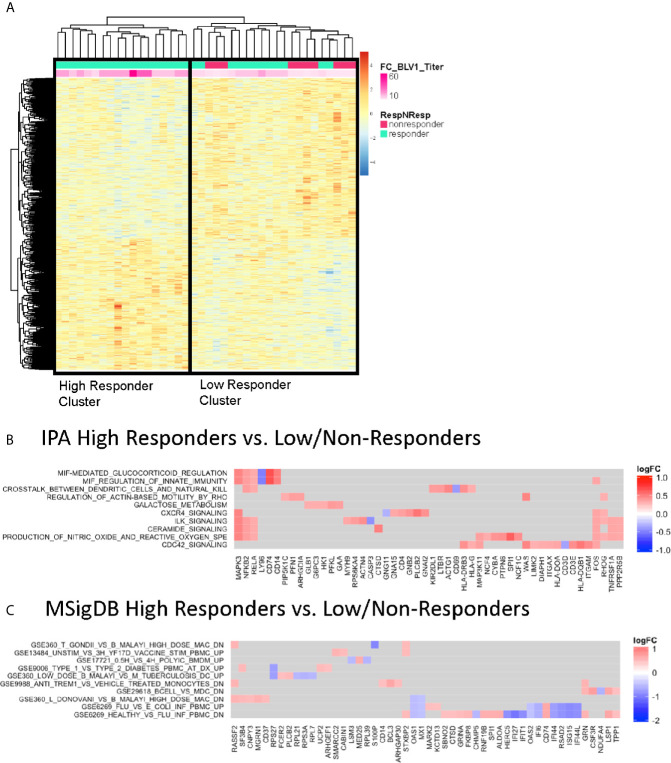
Correlates of Vaccine-induced Antibody Responses Post-Vaccination. **(A)** Heatmap representation of the top significantly correlated transcripts from regressing baseline expression from all participants and fold change difference in pH1N1 antibody titers at V1 compared to BL. The expression intensities are represented using a blue-white-red color scale. Rows correspond to probes and columns correspond to profiled samples (p ≤ 0.05). “High” responder and “low/non” responder clusters were compared and subjected to gene set enrichment analysis using the IPA database **(B)** and MSigDB Immunologic signature model **(C)**.

Given that pH1N1 was a novel antigen in the P1088 study cohort, participants received a boost at 21-28 days after the first vaccination. We investigated molecular signatures at this timepoint using differentially expressed gene profiles from PBMC at V1 prior to the boost vaccination. Regression analysis was performed and supervised based on the fold change in pH1N1 titer (V1/BL), as in **Figure 2** (top genes listed in **Supplementary Table S2**). This analysis also generated two clusters with one containing all non-responders and some low responders and the other with high responders (**Figure 3A**). IPA analysis of the top correlating genes revealed lower expression of the activation marker *CD69* (as a member of the ‘Crosstalk between Dendritic cells and Natural Killer cells’ pathway) and *LY96*, whose protein associates with TLR4 to respond to LPS in HR compared with LNR (**Figure 3B**). MSigDB analysis (**Figure 3C**) showed that expression of multiple IFN-inducible genes (*IFI16, IFI27, IFI44, IFI44L, IFIT1, ISG15, OAS1, OAS2, MX1*) were higher in LNR. These genes are upregulated in PBMC during acute viral and bacterial infections (GSE6269) (32).

### GSEA Using Cell-Type Specific Gene Database

**Figure 4:**
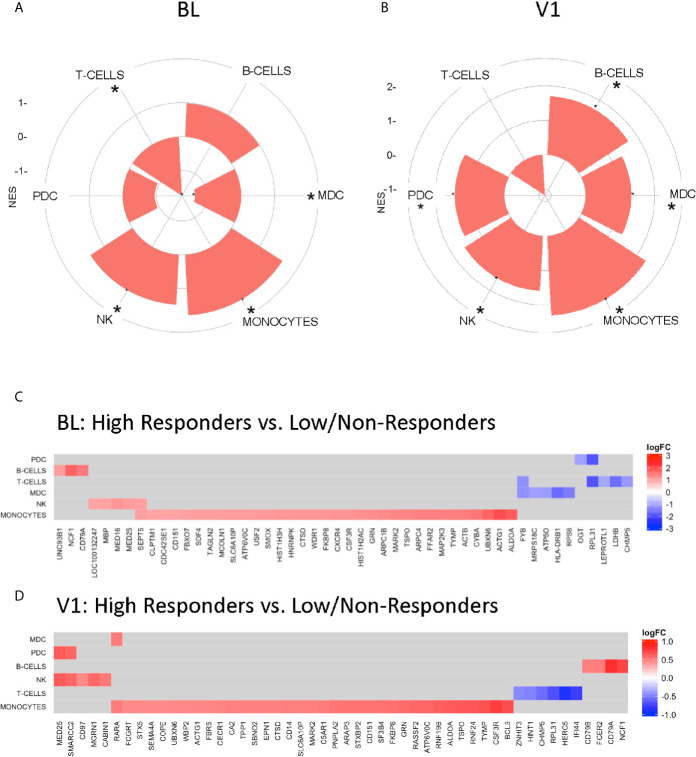
Gene Set Enrichment using Nakaya.NatImmunology Cell Specific Signatures. “High” responder and “low” responder clusters from regression analysis with baseline gene expression **(A**, **C)** and V1 gene expression **(B**, **D)** and fold change difference in pH1N1 titers (V1/BL) were subjected to gene set enrichment analysis using the Nakaya modules (21). **(A**, **B)** Radial plots illustrating selective enrichment in major PBMC cell types high responders compared to low/non-responders. **(C**, **D)** Genesets induced in a specific subset are significantly enriched (adjusted p-value <0.05 denoted by *) among genes upregulated or downregulated with respect to the enrichment score – (NES) between groups.

A caveat of systems biology approaches using whole blood samples is the inability to evaluate the contribution of specific cell populations to the observed transcriptomic profiles. We employed a cell-type specific database for gene set enrichment (21) to clusters identified in previous analysis that associated with Responder groups at each timepoint. Gene signatures related to B cells, NK, and monocytes were enriched in HR at BL, while DC subsets and T cells were enriched in LNR (**Figure 4A**). At V1, DC subsets were enriched in HR along with B cells, NK, and monocytes, while T cell signatures remained enriched in LNR (**Figure 4B**). Monocyte-associated gene expression accounted for most of the observed genes and showed enrichment at BL and V1 in HR (**Figures 4C, D**, respectively). This signature shared multiple genes (*ACTG1, ALDOA, ATP6V06, CD151, CSF3R, CTSD, FKBP8, GRN, MARK2, SLC6A10P, TSPO, TYMP, and UBXN6*) between the two timepoints (**Figure 4B**).

### Pathway Analysis by Age of Study Participants

**Figure 5:**
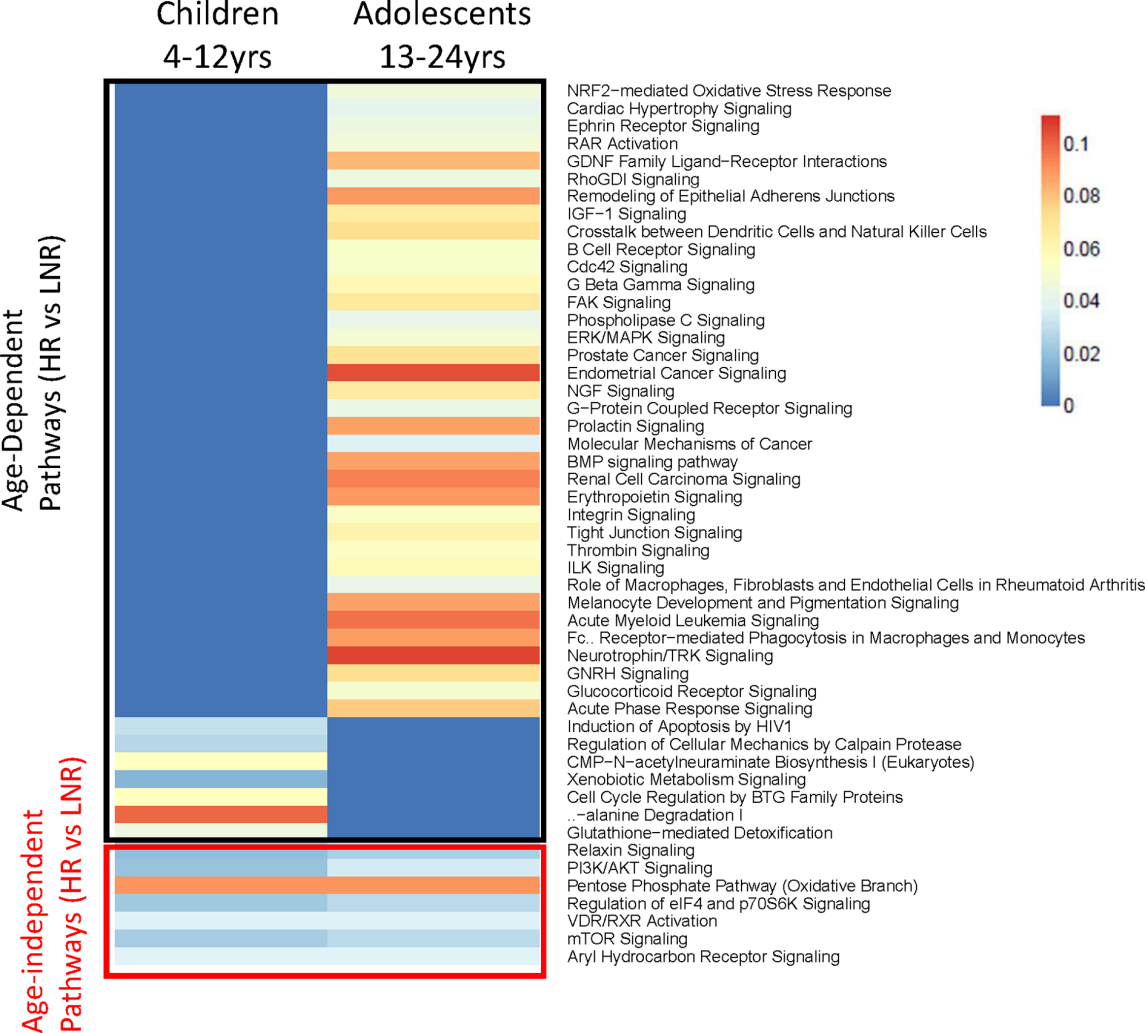
Pathway Analysis in two Age Groups at Baseline. Heatmap showing statistically significant canonical pathways (IPA) (both uniquely and commonly) regulated in high responders versus low/non-responders in the two age groups (4-12 years and 13-24 years) at baseline. Genes with an adjusted p-value <0.05, |FC|>1.3 and associated with canonical IPA pathway were used for analysis. Heat scaling refers to results from over-representation test performed using Fisher Exact Test (red indicating greater gene enrichment in the pathway). All pathways shown are statistically significant (p value <* *0.05) in one or both groups.

The P1088 study enrolled participants representing a broad age range from 4 to 24 years old, however age was not associated with Responder status (29). In the subset of participants analyzed by microarray, there was similarly no correlation between age and fold change of antibody titers (r=0.015). However, we reasoned that puberty may affect gene expression profiles in HIV-infected children and adolescents and therefore divided the donors into two age groups for further pathway analyses: 4-12 years (“children”; n=16) and 13-24 years (“adolescents”, n=24). For this analysis individuals were compared in each age group based on responder status: fold change of ≥ 4 were considered high responders (HR) and < 4 were considered low/non-responders (LNR). IPA analysis of gene expression at BL revealed a group of molecular pathways that were induced in both age groups (e.g. age-independent) as well as age-dependent pathways for each age group (**Figure 5**). Age-independent pathways enriched in HR were related to metabolic pathways (Pentose Phosphate-Oxidative branch, Aryl hydrocarbon receptor, Vitamin D/Retinoic acid receptor), and cell survival and protein synthesis pathways (PI3K/AKT, eIF4 and p70S6K, mTOR) confirming data from regression and cluster analysis in **Figure 2**. HR in the adolescent group demonstrated enrichment in more pathways than children including numerous pathways involved in cell growth (RAR activation, Cdc42, Rho, G beta gamma signaling), cell adhesion and mobility (remodeling of Epithelial Adherens Junctions, Integrin and Tight Junction signaling) and hormonal and growth factor signaling (prolactin, IGF-1, NGF, BMP and GNRH signaling).

**Figure 6:**
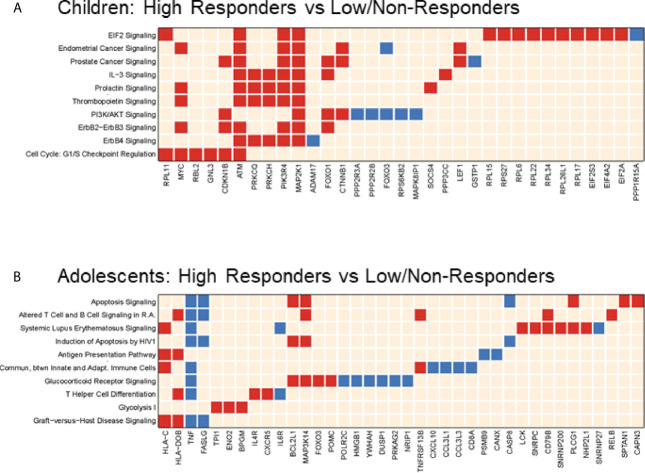
Pathways Analysis in two Age Groups at Post-Vaccination. Top 10 (or top selected) significant pathways and their gene members enriched when comparing high responder versus low/non-responder at Visit 1 in 4-12yr group **(A)** and 13-24yr group **(B)**. Each row is a regulated canonical pathway (ingenuity software); each column represents an up-(red) or down-(blue) regulated genes (p-value <= 0.05 and |FC|>1.3) induced in 1 or more pathways(s). Over representation test was performed using Fisher Exact Test; significance, displayed on the right, is achieved for p<0.05 (-log(p)1.3).

Consistent with BL data, HR in the younger age group exhibited distinct molecular signatures from the adolescent subset at V1, sharing only one gene; Forkhead box O3 (*FOXO3*) amongst the top 10 enriched pathways (**Figure 6**). Molecular pathways related to cell cycle and protein translation were enriched in children HR including EIF2 signaling, an indicator of ER stress and unfolded protein response (UPR) (**Figure 6A**). In the adolescent group, classic inflammatory markers such as *TNF*, *FASLG*, and *CXCL10* had higher expression in LNR compared to HR (**Figure 6B**). In the younger group, ‘classical’ inflammatory markers were not identified, however other inflammation-related genes such as *ADAM17, GSTP1*, and *PPP1R15A* were upregulated in LNR, suggesting that different, age-dependent mechanisms of inflammation may be responsible for poor influenza vaccine responses.

### Markers of T Follicular Helper Cells Are Enriched in High Responders to pH1N1 Vaccine

**Figure 7:**
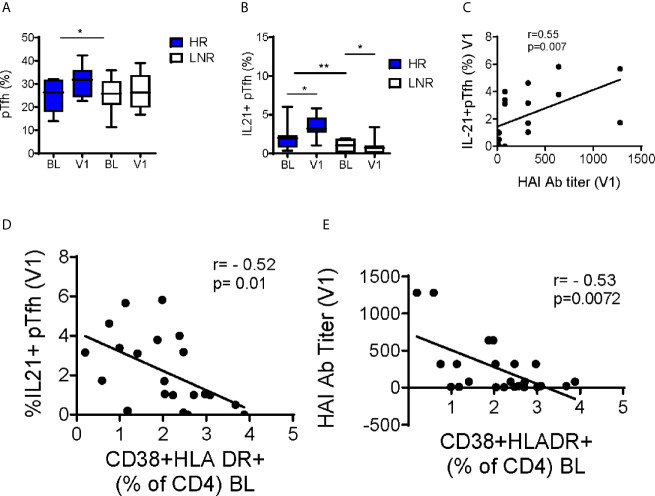
Increase in pTfh Frequency and Function Post-Vaccination in pH1N1 Responders. **(A)** Frequencies of CD4+CD45RO+CXCR5+ peripheral T follicular helper cells (pTfh) without stimulation and **(B)** IL-21+ pTfh after 12-hour stimulation with H1N1 from responders (n=14) and non-responders (n=14) at baseline (BL) and post vaccination (visit 1, V1). Pearson correlations between **(C)** IL-21+pTfh at V1 following H1N1 stimulation with HAI titers at V1 **(D)** IL-21+pTfh at V1 following H1N1 stimulation with baseline CD4 Immune activation **(E)** HAI titers at V1 with baseline CD4 Immune activation. P-values were calculated with Student’s t-test or Mann-Whitney test as appropriate. Box plots include median with 25th and 75th percentile borders, and error bars represent 10th and 90th percentiles. Stars indicate the level of significance: *p < 0.05, **p < 0.01.

Higher expression of the CXC chemokine receptor type 5 (*CXCR5*) was noted at V1 in HR (**Figure 6B**). CXCR5 is a homing marker of T follicular helper cells (Tfh), a CD4+ T cell subset essential for supporting B cell function and differentiation *via* abundant production of IL-21 in germinal centers (GC). A proportion of circulating CD4+ T cells express CXCR5 and exhibit functional properties of GC Tfh (33–35). To explore the significance of *CXCR5* expression in the microarray data in HR at V1, we investigated peripheral (pTfh) frequencies and function by flow cytometry. We did not observe differences in the frequency of pTfh prior to vaccination, however frequencies of pTfh (CD4+CD45RO+CXCR5+) were significantly higher at V1 compared to BL in HR only (**Figure 7A**). Upon *in vitro* stimulation of PBMC with pH1N1 antigen, pTfh from HR produced significantly more IL-21 at V1 compared to LNR and frequency of IL-2-producing pTfh positively correlated with HAI titer at V1 (**Figures 7B, C**, respectively).

To investigate the relationship immune activation and vaccine response, we measured co-expression of CD38 and HLA-DR on CD4+ T cells. At BL, LNR exhibited increased frequencies of CD38+HLADR+ CD4+ T cells compared to HR (2.5 +/-0.29 vs. 1.6 +/-0.26, respectively, p=0.04). CD38+HLADR+ CD4+ T cells at BL showed negative correlations with IL-21-producing pTfh and pH1N1 titer at V1 (**Figures 7D, E**, respectively). These data provide a link between known immunological correlates of influenza vaccine response to immunological and transcriptional signatures at pre-vaccination.

## Discussion

In this study, transcriptomic analyses of whole blood were applied to identify gene signatures related to novel pH1N1 vaccine responses in HIV-infected children and adolescents under suppressive ART. We used multiple GSEA platforms to generate a comprehensive resource of the transcriptomic changes related to antibody responses in young PWH. Our hypothesis was that chronic immune activation would influence vaccine responses (36). Previously, our group has shown that prior to vaccination markers of immune activation, including CD38+ HLA-DR+ T cells and serum levels of TNF and other inflammatory markers, negatively correlate with antibody responses to seasonal influenza vaccination (including pH1N1) in multiple cohorts of HIV-infected ART-treated adults (37–40). The current study in a pediatric cohort confirmed these observations as we demonstrated increased immune activation shown by negative correlations of the CD38+HLA-DR+ CD4 T cell frequencies with serum antibody titers, and this is further supported by GSEA showing DC and T cell related genes significantly enriched in LNR at pre-vaccination. Additionally, we observed higher CD69 following vaccination in LNR which is an early activation marker expressed on leukocytes (especially T and NK cells) and its dysregulation is associated with multiple inflammatory diseases (41).

Metabolic stress pathways (mitochondrial dysfunction and oxidative phosphorylation) were differentially expressed in transcriptional analysis at baseline in high and low/non-responders and this result was consistent regardless of whether participants were grouped by age. The link between metabolic programs and immune function have been described (42–44). Mitochondria have well-characterized roles in cellular energy and apoptosis and have been shown to play an important role in priming the innate immune system in the context of viral and bacterial infection (45). Our findings were supportive of this link with the observation that the potent anti-apoptotic protein, *BCL2L1*, was enriched in HNR at baseline and this gene has been shown to be regulated by mitochondrial transcription factor A (46). Moreover, ART is associated with numerous side effects, of which mitochondrial toxicity is one. The mechanisms of toxicity *in vivo* are unclear and controversial due to the use of multiple drugs and classes of drug by each patient and the reliance on *in vitro* data for determining drug effects on mitochondria (47). The question remains how alterations in mitochondrial function and quality relate to chronic immune activation and vaccine responses.

In the present study, GSEA using cell specific gene signatures confirmed that B cells were enriched post-vaccination in high responders, but our analysis did not identify them as a predictive cell-type (**Figure 3**). UPR is upregulated in plasmablasts in order to support high levels of antibody production (48, 49), therefore this signature may represent an ongoing or residual antibody response in responders to pH1N1 vaccination. Typically, the plasmablast response to influenza vaccination peaks at 7 days (50), however given that pH1N1 was a novel antigen in the participant group it is possible the response was delayed to remain detectable 3 weeks post-infection. The B cell compartment is highly heterogeneous and the methodology used here was not sensitive enough to detect a rare predictive subset however, peripheral T follicular helper (pTfh) cells have been shown to correlate with vaccine-induced antibody responses in HIV-infected and HIV negative populations. Specifically, IL-21 producing pTfh are a strong immunological correlate of T-dependent B cell responses against influenza antigens (51, 52), as well as HIV (53) and malaria vaccine antigens (54).

Monocyte signatures were significantly enriched before and after vaccination and may provide a target for predicting vaccine effectiveness. Indeed, pre-vaccination expression of costimulatory molecules, CD80 and CD86 on TLR-activated monocytes from elderly and young HIV-uninfected adults was shown to associate with vaccine responses to influenza (55). Our results did not address monocyte function since samples were analyzed without stimulus; however, we found increased IFN-inducible gene expression in LNR at V1 by multiple analysis platforms (IPA, MSigDB, and Age-specific) which could be attributed to the monocyte population. Proteins encoded by IFN-inducible genes are essential antiviral effectors with capabilities to block at various steps of the viral life cycle (56), however unregulated IFN responses can lead to immune dysfunction. The role of type I IFN in HIV infection is complex; it is important in controlling viral replication very early following infection while contributing to pathogenesis in chronic infection (57). Because the difference in IFN-inducible gene expression was not present prior to vaccination, our findings beg the question of what effect routine vaccination is having on viral replication and the existing HIV infection. Influenza vaccination has been shown to increase plasma viremia transiently following vaccination (peak 2 weeks post-vaccination) (58). Future studies monitoring vaccine responses in PWH on ART should consider a possible effect on viral recrudescence. it will be important to evaluate the role of monocytes in influencing antibody-driven vaccine responses. *CSF3R* encodes the receptor for granulocyte-colony stimulating factor (G-CSF) and is expressed on circulating, classical monocytes (CD14+CD16-) (59) suggesting that enrichment of this predominant monocyte subset could be an immunological correlate for antibody responses as well as a biomarker or predictor of response.

Despite the intriguing findings in this cohort there were several limitations inherent in the present study. The study design catered to blood sampling coincident with important timepoints for measuring serum antibody responses. The inclusion of blood sampling at an early timepoint post-vaccination (e.g. day 2-7) would have allowed for evaluation of the innate immune response to the vaccine, however the innate response to seasonal influenza vaccine (trivalent-inactivated) has been studied extensively in HIV-uninfected populations (18, 21, 22, 60, 61). Thus, our study is focused on creating a resource of later adaptive immune biomarkers that correlate with serum antibody titers to a novel influenza antigen and, therefore, response. As one of the few transcriptomic studies in perinatal HIV infected children and adolescents, the data presented herein will undoubtedly serve as a novel resource for further immune monitoring studies in this population and may especially be important in light of the current pandemic SARS-CoV-2.

Unexpectedly, this cohort had relatively high baseline antibody responses to pH1N1 despite no documented exposure to the antigen (29), while other cohorts with participants at similar ages displayed low baseline titers and a lack of cross-reactivity (62). Despite the complexity in using systems biology to predict immunogenicity to influenza vaccines due to individual infection and vaccination histories, we believe that our transcriptomic study in young PWH fills an age gap and has yielded a valuable resource likely to provide insight into favorable and negative targets for improving vaccine design and assessment of vaccine responses in young PWH subjects.

## AUTHOR'S NOTE

Parts of the work described in this manuscript have been presented at Conference of Retrovirus and Opportunistic Infections 2013 and American Association of Immunologists 2014 as poster presentations by A.P. and L.D., respectively.

## Data Availability Statement

The data presented in the study are deposited in the Gene Expression Omnibus data repository (https://www.ncbi.nlm.nih.gov/geo/) under the accession number GSE167893. 

## Ethics Statement

The studies involving human participants were reviewed and approved by International Maternal Pediatric Adolescent AIDS Clinical Trials Network. Written informed consent to participate in this study was provided by the participants’ legal guardian/next of kin.

## Author Contributions

SP, AW, CC, R-PS, and MC were involved in conception of the work. CS, AP, VG, and AF-M were involved in data collection. LD, CS, AP, VG, AF-M, R-PS, MC, and SP, data analysis and interpretation. LD, MC, and SP drafted the article. LD, CS, VG, AW, CC, R-PS, MC, and SP provided critical revision of the article. All authors contributed to the article and approved the submitted version.

## Funding

This work was funded by an ARRA IMPACT grant to SP. Overall support for the International Maternal Pediatric Adolescent AIDS Clinical Trials Group (IMPAACT) was provided by the National Institute of Allergy and Infectious Diseases (NIAID) [U01 AI068632], the Eunice Kennedy Shriver National Institute of Child Health and Human Development (NICHD) and the National Institute of Mental Health (NIMH) [AI068632]. This work was supported by the Statistical and Data Analysis Center at Harvard School of Public Health, under the National Institute of Allergy and Infectious Diseases cooperative agreement #5 U01 AI41110 with the Pediatric AIDS Clinical Trials Group (PACTG) and #1 U01 AI068616 with the IMPAACT Group. Support of the sites was provided by the National Institute of Allergy and Infectious Diseases (NIAID) and the NICHD International and Domestic Pediatric and Maternal HIV Clinical Trials Network funded by NICHD (contract number N01-DK-9–001/HHSN267200800001C). This publication was made possible by support for the Miami Center for AIDS Research (CFAR) at the University of Miami Miller School of Medicine funded by a grant (P30AI073961) and to SP (R01AI108472) from the National Institutes of Health (NIH) and R01, which is supported by the following NIH Co-Funding and Participating Institutes and Centers: NIAID, NCI, NICHD, NHLBI, NIDA, NIMH, NIA, NIDDK, NIGMS, FIC AND OAR. The content is solely the responsibility of the authors and does not necessarily represent the official views of the National Institutes of Health.

## Conflict of Interest

The authors declare that the research was conducted in the absence of any commercial or financial relationships that could be construed as a potential conflict of interest.

## References

[1] Sheth AN Althoff KN Brooks JT. Influenza susceptibility, severity, and shedding in HIV-infected adults: a review of the literature. Clin Infect Dis (2011) 52(2):219–27. 10.1093/cid/ciq110 PMC4990828 21288848

[2] Remschmidt C Wichmann O Harder T. Influenza vaccination in HIV-infected individuals: systematic review and assessment of quality of evidence related to vaccine efficacy, effectiveness and safety. Vaccine (2014) 32(43):5585–92. 10.1016/j.vaccine.2014.07.101 25131742

[3] Grohskopf LA Alyanak E Broder KR Walter EB Fry AM Jernigan DB. Prevention and Control of Seasonal Influenza with Vaccines: Recommendations of the Advisory Committee on Immunization Practices - United States, 2019-20 Influenza Season. MMWR Recomm Rep (2019) 68(3):1–21. 10.15585/mmwr.rr6803a1 PMC6713402 31441906

[4] Shang M Chung JR Jackson ML Jackson LA Monto AS Martin ET. Influenza vaccine effectiveness among patients with high-risk medical conditions in the United States, 2012-2016. Vaccine (2018) 36(52):8047–53. 10.1016/j.vaccine.2018.10.093 PMC6282182 30420119

[5] Frasca D Diaz A Romero M Mendez NV Landin AM Blomberg BB. Effects of age on H1N1-specific serum IgG1 and IgG3 levels evaluated during the 2011-2012 influenza vaccine season. Immun Ageing (2013) 10(1):14. 10.1186/1742-4933-10-14 23607926 PMC3639840

[6] McElhaney JE. Influenza vaccine responses in older adults. Ageing Res Rev (2011) 10(3):379–88. 10.1016/j.arr.2010.10.008 PMC3061971 21055484

[7] Pallikkuth S De Armas LR Pahwa R Rinaldi S George VK Sanchez CM. Impact of aging and HIV infection on serologic response to seasonal influenza vaccination. AIDS (2018) 32(9):1085–94. 10.1097/QAD.0000000000001774 PMC6574117 29424779

[8] Abzug MJ Song LY Fenton T Nachman SA Levin MJ Rosenblatt HM. Pertussis booster vaccination in HIV-infected children receiving highly active antiretroviral therapy. Pediatrics (2007) 120(5):e1190–202. 10.1542/peds.2007-0729 17938165

[9] Obaro SK Pugatch D Luzuriaga K. Immunogenicity and efficacy of childhood vaccines in HIV-1-infected children. Lancet Infect Dis (2004) 4(8):510–8. 10.1016/S1473-3099(04)01106-5 15288824

[10] Vigano A Zuccotti GV Pacei M Erba P Castelletti E Giacomet V. Humoral and cellular response to influenza vaccine in HIV-infected children with full viroimmunologic response to antiretroviral therapy. J Acquir Immune Defic Syndr (2008) 48(3):289–96. 10.1097/QAI.0b013e3181632cda 18545155

[11] Krammer F. The human antibody response to influenza A virus infection and vaccination. Nat Rev Immunol (2019) 19(6):383–97. 10.1038/s41577-019-0143-6 30837674

[12] Wang TT Bournazos S Ravetch JV. Immunological responses to influenza vaccination: lessons for improving vaccine efficacy. Curr Opin Immunol (2018) 53:124–9. 10.1016/j.coi.2018.04.026 PMC6141319 29753885

[13] Thomas PG Keating R Hulse-Post DJ Doherty PC. Cell-mediated protection in influenza infection. Emerg Infect Dis (2006) 12(1):48–54. 10.3201/eid1201.051237 16494717 PMC3291410

[14] Ohmit SE Petrie JG Cross RT Johnson E Monto AS. Influenza hemagglutination-inhibition antibody titer as a correlate of vaccine-induced protection. J Infect Dis (2011) 204(12):1879–85. 10.1093/infdis/jir661 21998477

[15] Querec TD Akondy RS Lee EK Cao W Nakaya HI Teuwen D. Systems biology approach predicts immunogenicity of the yellow fever vaccine in humans. Nat Immunol (2009) 10(1):116–25. 10.1038/ni.1688 PMC4049462 19029902

[16] Caskey R Lindau ST Alexander GC. Knowledge and early adoption of the HPV vaccine among girls and young women: results of a national survey. J Adolesc Health (2009) 45(5):453–62. 10.1016/j.jadohealth.2009.04.021 19837351

[17] Gaucher D Therrien R Kettaf N Angermann BR Boucher G Filali-Mouhim A. Yellow fever vaccine induces integrated multilineage and polyfunctional immune responses. J Exp Med (2008) 205(13):3119–31. 10.1084/jem.20082292 PMC2605227 19047440

[18] Li S Rouphael N Duraisingham S Romero-Steiner S Presnell S Davis C. Molecular signatures of antibody responses derived from a systems biology study of five human vaccines. Nat Immunol (2014) 15(2):195–204. 10.1038/ni.2789 24336226 PMC3946932

[19] O’Connor D Pinto MV Sheerin D Tomic A Drury RE Channon-Wells S. Gene expression profiling reveals insights into infant immunological and febrile responses to group B meningococcal vaccine. Mol Syst Biol (2020) 16(11):e9888. 10.15252/msb.20209888 33210468 PMC7674973

[20] Obermoser G Presnell S Domico K Xu H Wang Y Anguiano E. Systems scale interactive exploration reveals quantitative and qualitative differences in response to influenza and pneumococcal vaccines. Immunity (2013) 38(4):831–44. 10.1016/j.immuni.2012.12.008 PMC3681204 23601689

[21] Nakaya HI Wrammert J Lee EK Racioppi L Marie-Kunze S Haining WN. Systems biology of vaccination for seasonal influenza in humans. Nat Immunol (2011) 12(8):786–95. 10.1038/ni.2067 PMC3140559 21743478

[22] Tan Y Tamayo P Nakaya H Pulendran B Mesirov JP Haining WN. Gene signatures related to B-cell proliferation predict influenza vaccine-induced antibody response. Eur J Immunol (2014) 44(1):285–95. 10.1002/eji.201343657 PMC3973429 24136404

[23] Tomic A Tomic I Rosenberg-Hasson Y Dekker CL Maecker HT Davis MM. SIMON, an Automated Machine Learning System, Reveals Immune Signatures of Influenza Vaccine Responses. J Immunol (2019) 203(3):749–59. 10.4049/jimmunol.1900033 PMC6643048 31201239

[24] Cox RJ Brokstad KA Zuckerman MA Wood JM Haaheim LR Oxford JS. An early humoral immune response in peripheral blood following parenteral inactivated influenza vaccination. Vaccine (1994) 12(11):993–9. 10.1016/0264-410X(94)90334-4 7975853

[25] el-Madhun AS RJ C Soreide A Olofsson J Haaheim LR. Systemic and mucosal immune responses in young children and adults after parenteral influenza vaccination. J Infect Dis (1998) 178(4):933–9. 10.1086/515656 9806018

[26] Sasaki S He XS Holmes TH Dekker CL Kemble GW Arvin AM. Influence of prior influenza vaccination on antibody and B-cell responses. PloS One (2008) 3(8):e2975. 10.1371/journal.pone.0002975 18714352 PMC2500171

[27] Team H-CSP Consortium H-I. Multicohort analysis reveals baseline transcriptional predictors of influenza vaccination responses. Sci Immunol (2017) 2(14). 10.1126/sciimmunol.aal4656 PMC5800877 28842433

[28] Chen H Wang Y Liu W Zhang J Dong B Fan X. Serologic survey of pandemic (H1N1) 2009 virus, Guangxi Province, China. Emerg Infect Dis (2009) 15(11):1849–50. 10.3201/eid1511.090868 PMC2857250 19891883

[29] Flynn PM Nachman S Muresan P Fenton T Spector SA Cunningham CK. Safety and immunogenicity of 2009 pandemic H1N1 influenza vaccination in perinatally HIV-1-infected children, adolescents, and young adults. J Infect Dis (2012) 206(3):421–30. 10.1093/infdis/jis360 PMC3490699 22615311

[30] Levin MJ Song LY Fenton T Nachman S Patterson J Walker R. Shedding of live vaccine virus, comparative safety, and influenza-specific antibody responses after administration of live attenuated and inactivated trivalent influenza vaccines to HIV-infected children. Vaccine (2008) 26(33):4210–7. 10.1016/j.vaccine.2008.05.054 PMC2615200 18597900

[31] Pallikkuth S Pilakka Kanthikeel S Silva SY Fischl M Pahwa R Pahwa S. Upregulation of IL-21 receptor on B cells and IL-21 secretion distinguishes novel 2009 H1N1 vaccine responders from nonresponders among HIV-infected persons on combination antiretroviral therapy. J Immunol (2011) 186(11):6173–81. 10.4049/jimmunol.1100264 PMC3170914 21531891

[32] Ramilo O Allman W Chung W Mejias A Ardura M Glaser C. Gene expression patterns in blood leukocytes discriminate patients with acute infections. Blood (2007) 109(5):2066–77. 10.1182/blood-2006-02-002477 PMC1801073 17105821

[33] Morita R Schmitt N Bentebibel SE Ranganathan R Bourdery L Zurawski G. Human blood CXCR5(+)CD4(+) T cells are counterparts of T follicular cells and contain specific subsets that differentially support antibody secretion. Immunity (2011) 34(1):108–21. 10.1016/j.immuni.2010.12.012 PMC3046815 21215658

[34] Fraietta JA Mueller YM Yang G Boesteanu AC Gracias DT Do DH. Type I interferon upregulates Bak and contributes to T cell loss during human immunodeficiency virus (HIV) infection. PloS Pathog (2013) 9(10):e1003658. 10.1371/journal.ppat.1003658 24130482 PMC3795023

[35] Locci M Havenar-Daughton C Landais E Wu J Kroenke MA Arlehamn CL. Human circulating PD-1+CXCR3-CXCR5+ memory Tfh cells are highly functional and correlate with broadly neutralizing HIV antibody responses. Immunity (2013) 39(4):758–69. 10.1016/j.immuni.2013.08.031 PMC3996844 24035365

[36] Paiardini M Muller-Trutwin M. HIV-associated chronic immune activation. Immunol Rev (2013) 254(1):78–101. 10.1111/imr.12079 23772616 PMC3729961

[37] Parmigiani A Alcaide ML Freguja R Pallikkuth S Frasca D Fischl MA. Impaired antibody response to influenza vaccine in HIV-infected and uninfected aging women is associated with immune activation and inflammation. PloS One (2013) 8(11):e79816. 10.1371/journal.pone.0079816 24236161 PMC3827419

[38] de Armas LR Pallikkuth S George V Rinaldi S Pahwa R Arheart KL. Reevaluation of immune activation in the era of cART and an aging HIV-infected population. JCI Insight (2017) 2(20). 10.1172/jci.insight.95726 PMC5846952 29046481

[39] George VK Pallikkuth S Parmigiani A Alcaide M Fischl M Arheart KL. HIV infection Worsens Age-Associated Defects in Antibody Responses to Influenza Vaccine. J Infect Dis (2015) 211(12):1959–68. 10.1093/infdis/jiu840 PMC4836723 25556252

[40] Pallikkuth S Parmigiani A Silva SY George VK Fischl M Pahwa R. Impaired peripheral blood T-follicular helper cell function in HIV-infected nonresponders to the 2009 H1N1/09 vaccine. Blood (2012) 120(5):985–93. 10.1182/blood-2011-12-396648 PMC3412336 22692510

[41] Gonzalez-Amaro R Cortes JR Sanchez-Madrid F Martin P. Is CD69 an effective brake to control inflammatory diseases? Trends Mol Med (2013) 19(10):625–32. 10.1016/j.molmed.2013.07.006 PMC4171681 23954168

[42] Domblides C Lartigue L Faustin B. Metabolic Stress in the Immune Function of T Cells, Macrophages and Dendritic Cells. Cells (2018) 7(7):68. 10.3390/cells7070068 PMC6070887 29966302

[43] Marijt KA Sluijter M Blijleven L Tolmeijer SH Scheeren FA van der Burg SH. Metabolic stress in cancer cells induces immune escape through a PI3K-dependent blockade of IFNgamma receptor signaling. J Immunother Cancer (2019) 7(1):152. 10.1186/s40425-019-0627-8 31196219 PMC6567539

[44] Schoeman JC Moutloatse GP Harms AC Vreeken RJ Scherpbier HJ Van Leeuwen L. Fetal Metabolic Stress Disrupts Immune Homeostasis and Induces Proinflammatory Responses in Human Immunodeficiency Virus Type 1- and Combination Antiretroviral Therapy-Exposed Infants. J Infect Dis (2017) 216(4):436–46. 10.1093/infdis/jix291 PMC5853663 28633455

[45] West AP Khoury-Hanold W Staron M Tal MC Pineda CM Lang SM. Mitochondrial DNA stress primes the antiviral innate immune response. Nature (2015) 520:553–7. 10.1016/j.bpj.2014.11.029 PMC4409480 25642965

[46] Kurita T Izumi H Kagami S Kawagoe T Toki N Matsuura Y. Mitochondrial transcription factor A regulates BCL2L1 gene expression and is a prognostic factor in serous ovarian cancer. Cancer Sci (2012) 103(2):239–444. 10.1111/j.1349-7006.2011.02156.x 22098591

[47] Apostolova N Blas-Garcia A Esplugues JV. Mitochondrial interference by anti-HIV drugs: mechanisms beyond Pol-gamma inhibition. Trends Pharmacol Sci (2011) 32(12):715–25. 10.1016/j.tips.2011.07.007 21899897

[48] Gass JN Gifford NM Brewer JW. Activation of an unfolded protein response during differentiation of antibody-secreting B cells. J Biol Chem (2002) 277(50):49047–54. 10.1074/jbc.M205011200 12374812

[49] Iwakoshi NN Lee AH Vallabhajosyula P Otipoby KL Rajewsky K Glimcher LH. Plasma cell differentiation and the unfolded protein response intersect at the transcription factor XBP-1. Nat Immunol (2003) 4(4):321–9. 10.1038/ni907 12612580

[50] Davis CW Jackson KJL McCausland MM Darce J Chang C Linderman SL. Influenza vaccine-induced human bone marrow plasma cells decline within a year after vaccination. Science (2020) 370(6513):237–41. 10.1126/science.aaz8432 PMC10155619 32792465

[51] de Armas LR Cotugno N Pallikkuth S Pan L Rinaldi S Sanchez MC. Induction of IL21 in Peripheral T Follicular Helper Cells Is an Indicator of Influenza Vaccine Response in a Previously Vaccinated HIV-Infected Pediatric Cohort. J Immunol (2017) 198(5):1995–2005. 10.4049/jimmunol.1601425 28130496 PMC5322168

[52] Pallikkuth S de Armas LR Rinaldi S George VK Pan L Arheart KL. Dysfunctional peripheral T follicular helper cells dominate in people with impaired influenza vaccine responses: Results from the FLORAH study. PloS Biol (2019) 17(5):e3000257. 10.1371/journal.pbio.3000257 31100059 PMC6542545

[53] Schultz BT Teigler JE Pissani F Oster AF Kranias G Alter G. Circulating HIV-Specific Interleukin-21(+)CD4(+) T Cells Represent Peripheral Tfh Cells with Antigen-Dependent Helper Functions. Immunity (2016) 44(1):167–78. 10.1016/j.immuni.2015.12.011 26795249

[54] Pallikkuth S Chaudhury S Lu P Pan L Jongert E Wille-Reece U. A delayed fractionated dose RTS,S AS01 vaccine regimen mediates protection via improved T follicular helper and B cell responses. Elife (2020) 9:e51889. 10.7554/eLife.51889 32342859 PMC7213985

[55] van Duin D Allore HG Mohanty S Ginter S Newman FK Belshe RB. Prevaccine determination of the expression of costimulatory B7 molecules in activated monocytes predicts influenza vaccine responses in young and older adults. J Infect Dis (2007) 195(11):1590–7. 10.1086/516788 17471428

[56] Sadler AJ Williams BR. Interferon-inducible antiviral effectors. Nat Rev Immunol (2008) 8(7):559–68. 10.1038/nri2314 PMC2522268 18575461

[57] Sandler NG Bosinger SE Estes JD Zhu RT Tharp GK Boritz E. Type I interferon responses in rhesus macaques prevent SIV infection and slow disease progression. Nature (2014) 511(7511):601–5. 10.1038/nature13554 PMC4418221 25043006

[58] Staprans SI Hamilton BL Follansbee SE Elbeik T Barbosa P Grant RM. Activation of virus replication after vaccination of HIV-1-infected individuals. J Exp Med (1995) 182(6):1727–37. 10.1084/jem.182.6.1727 PMC2192265 7500017

[59] Mobley JL Leininger M Madore S Baginski TJ Renkiewicz R. Genetic evidence of a functional monocyte dichotomy. Inflammation (2007) 30(6):189–97. 10.1007/s10753-007-9036-0 17587162

[60] Nakaya HI Clutterbuck E Kazmin D Wang L Cortese M Bosinger SE. Systems biology of immunity to MF59-adjuvanted versus nonadjuvanted trivalent seasonal influenza vaccines in early childhood. Proc Natl Acad Sci USA (2016) 113(7):1853–8. 10.1073/pnas.1519690113 PMC4763735 26755593

[61] Nakaya HI Hagan T Duraisingham SS Lee EK Kwissa M Rouphael N. Systems Analysis of Immunity to Influenza Vaccination across Multiple Years and in Diverse Populations Reveals Shared Molecular Signatures. Immunity (2015) 43(6):1186–98. 10.1016/j.immuni.2015.11.012 PMC4859820 26682988

[62] Tsang JS Schwartzberg PL Kotliarov Y Biancotto A Xie Z Germain RN. Global analyses of human immune variation reveal baseline predictors of postvaccination responses. Cell (2014) 157(2):499–513. 10.1016/j.cell.2014.03.031 24725414 PMC4139290

